# Administration of gamma‐hydroxybutyrate instead of beta‐hydroxybutyrate to a liver transplant recipient suffering from propionic acidemia and cardiomyopathy: A case report on a medication prescribing error

**DOI:** 10.1002/jmd2.12090

**Published:** 2020-01-03

**Authors:** Caroline Tuchmann‐Durand, Eloise Thevenet, Florence Moulin, Fabrice Lesage, Juliette Bouchereau, Mehdi Oualha, Diala Khraiche, Anaïs Brassier, Camille Wicker, Stéphanie Gobin‐Limballe, Jean‐Baptiste Arnoux, Florence Lacaille, Clotilde Wicart, Bruno Coat, Joel Schlattler, Salvatore Cisternino, Sylvain Renolleau, Philippe‐Henri Secretan, Pascale De Lonlay

**Affiliations:** ^1^ Imagine Institut des Maladies Génétiques, Paris, France and Biotherapy Department Necker Children's Hospital, Assistance Publique‐Hôpitaux de Paris Paris France; ^2^ Reference Center for Inherited Metabolic Diseases Necker Children's Hospital, Assistance Publique‐Hôpitaux de Paris Paris France; ^3^ Intensive Care Unit Necker Children's Hospital, Assistance Publique‐Hôpitaux de Paris, Paris and Paris Descartes University Paris France; ^4^ Cardiology Care Unit Necker Children's Hospital, Assistance Publique‐Hôpitaux de Paris Paris France; ^5^ Molecular Genetic Department Necker Children's Hospital, Assistance Publique‐Hôpitaux de Paris Paris France; ^6^ Paediatric Hepatology Unit, Reference Center for Rare Pediatric Liver Diseases, Department of Gastroenterology‐Hepatology‐Nutrition Necker Children's Hospital, Assistance Publique‐Hôpitaux de Paris, Paris, APHP, Filière Filfoie, ERN Transplantchild Paris France; ^7^ Pharmacy's Department Necker Children's Hospital, Assistance Publique‐Hôpitaux de Paris Paris France; ^8^ Imagine Institut des Maladies Génétiques Filière G2M, MetabERN, INEM 1151, Paris Descartes University Paris France

**Keywords:** computerized prescription system, inborn error of metabolism, pharmacovigilance, orphan drug, ketone body, medication error, propionic

## Abstract

Beta‐hydroxybutyrate (BHB) is a synthetic ketone body used as an adjuvant energy substrate in the treatment of patients with metabolic cardiomyopathy. A medication prescribing error led to the administration of the general anesthetic sodium gamma‐hydroxybutyrate (GHB) instead of sodium BHB in a liver transplant recipient with propionic acidemia and cardiomyopathy, causing acute coma. A 15‐year‐old boy suffering from neonatal propionic acidemia underwent liver transplantation (LT) for metabolic decompensation and cardiomyopathy (treated with cardiotropic drugs and BHB) diagnosed a year previously. The patient had been rapidly extubated after LT, and was recovering well. Eight days after LT, the patient suddenly became comatose. No metabolic, immunological, hypertensive, or infectious complications were apparent. The brain magnetic resonance imaging and electroencephalography results were normal. The coma was soon attributed to a medication prescribing error: administration of GHB instead of BHB on day 8 post‐LT. The patient recovered fully within a few hours of GHB withdrawal. The computerized prescription system had automatically suggested the referenced anesthetic GHB (administered intravenously) instead of the non‐referenced ketone body BHB, triggering coma in our patient. A computerized prescription system generated a medication prescribing error for a rare disease, in which the general anesthetic GHB was mistaken for the nonreferenced energy substrate BHB.

**SYNOPSIS**

When treating patients with rare inborn errors of metabolism, healthcare professionals should be aware of the risk of GHB/BHB confusion in their medication prescription system and during clinical care.

## 1 INTRODUCTION

Ketone bodies (such as beta‐hydroxybutyrate (BHB), acetoacetate, and acetone) are produced from acetyl‐CoA (derived from fatty acid oxidation) in the liver, and constitute the main energy substrates for ketolysis in tissues. When fatty acid or glucose metabolism is impaired, ketone bodies constitute an alternative source of acetyl‐CoA by stimulating pyruvate carboxylation and tricarboxylic acid cycle anaplerosis.^1^ Accordingly, a racemic mixture of d,l‐BHB is reportedly an effective treatment for the cardiomyopathy observed in several energy deficiency diseases (such as multiple acyl‐CoA dehydrogenase deficiency and glycogen storage,^2^ also known as glycogen storage disease type III^3^) and for the prevention of neurological distress in pyruvate dehydrogenase deficiency.^4^

Propionic acidemia (PA) is a severe inborn error of metabolism caused by a defective propionyl‐CoA carboxylase (PCC) enzyme in the liver and other tissues.^5^ This mitochondrial matrix enzyme converts propionyl‐CoA into methylmalonyl‐CoA, which is then transformed into succinyl‐CoA. The latter subsequently enters the tricarboxylic acid cycle and generates components used by the mitochondrial respiratory chain. Hence, a PCC deficiency leads to the accumulation of propionyl‐CoA and its metabolites in the mitochondria, and impairment of the tricarboxylic acid cycle. Even when PA is treated aggressively, complications still occur; these notably include cardiomyopathy^6^ due to secondary dysfunction of the tricarboxylic acid cycle or the toxicity of propionyl‐CoA's metabolites (such as 3‐hydroxypropionate).^7^, ^8^ Liver transplantation (LT) has been developed as a treatment option in PA, in order to increase levels of enzyme activity, reduce the risk of metabolic decompensation, and improve cardiac function. Furthermore, Baruteau et al have suggested that BHB can reverse the cardiomyopathy seen in PA by supplying the heart with its preferred energy substrate (along with ATP).^9^

The endogenous compounds BHB and gamma‐hydroxybutyrate (GHB) are chemical isomers but differ in their pharmaceutical formulations and clinical effects. BHB is a synthetic ketone body that is formulated as a capsule in hospital immediately prior to use (at a dose of between 400 and 800 mg/kg per day) as an adjuvant energy substrate in cases of metabolic cardiomyopathy. In contrast, GHB (a precursor of the neurotransmitter GABA) induces central nervous system inhibition, narcolepsy and respiratory drive inhibition.^10^ However, GHB is abused recreationally as a “party drug” or “club drug,” with a wide array of formulations, patterns of use, and health risks.^11^, ^12^ In a clinical context, commercially available solutions for injection of GHB are used as a general anesthetic, thanks to the rapid induction sedation and amnesia. Furthermore, sodium oxybate (an oral solution of sodium GHB) is used clinically to treat adult patients suffering from narcolepsy with catalepsy.

**Figure 1:**
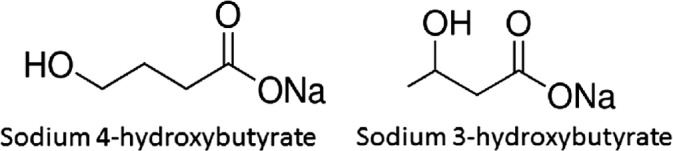
Chemical structures of sodium gamma‐hydroxybutyrate (GHB) and sodium beta‐hydroxybutyrate (BHB)

As isomers, sodium GHB and sodium BHB have the same chemical formula (C_4_H_7_NaO_3_) but differ with regard to the position of hydroxyl substitution on the butyrate backbone (Figure 1), and thus may be easily confused. Since PA is a rare disorder affecting a small number of patients, healthcare professionals may not be fully aware of this disease, its treatment, and associated pharmacovigilance issues.

Here, we report on a patient report with PA who required LT for multiple decompensations and cardiomyopathy, despite conventional treatment with cardiotropic drugs and BHB. After the LT, GHB was administered after it had been confused with BHB.

## 2 CASE REPORT

A 15‐year‐old boy underwent LT for PA complicated by cardiomyopathy. Propionic acidemia had been diagnosed at birth, since the patient presented the typical clinical features of neonatal coma at day 3 and ketoacidosis. After hemofiltration, the boy was treated with a low‐protein diet, an amino acid mixture, l‐carnitine, and enteral feeding. Propionic acidemia was diagnosed on the basis of (a) elevated blood and urine levels of specific propionate metabolites and (b) a homozygous mutation in the *PCCB* gene (c.990dupT). Over the course of the disease, the patient experienced six episodes of severe metabolic decompensations requiring hospitalisation in a critical care unit. Hemofiltration was required at the age of 3 months and again at the age of 4 years, and mechanical ventilation was required for pneumonia at the age of 2 years. In neurological terms, the patient suffered from visual and spatial dyspraxia, learning disability, and frontal lobe epilepsy. The latter condition had been under control since the age of 12. Hypokinetic dilated cardiomyopathy was diagnosed during an echocardiographic assessment at the age of 14 (left ventricular ejection fraction: 45%), and led to the prescription of cardiotropic drugs and BHB at a dose of 400 mg/kg/day and then 800 mg/kg/day. This treatment had no effect on the patient's cardiac function. At the age of 15, the patient received a liver transplant from a cadaver donor.

Although the patient had been rapidly extubated and was recovering well from surgery, he suddenly became comatose on day 8 post‐LT. No metabolic, immunological, or infectious complications were apparent, and the electroencephalography and brain MRI results were normal (data not shown). By checking all the prescriptions in the intensive care unit, the medical team discovered that on day 8 post‐LT, the patient had been treated with a sodium GHB solution for injection (Gamma‐OH 200 mg/mL, Serb, France) rather than the extemporaneous formulation of BHB previously received for PA—thus bringing about the coma. In fact, the computerized prescription system had automatically suggested GHB instead of the nonreferenced BHB. A pharmacovigilance report on this medication error was sent to the French Medicines Agency (*Agence nationale de sécurité du médicament et des produits de santé*). The patient recovered a few hours after the GHB had been withdrawn. He did not present any of the side effects sometimes associated with GHB administration (eg, hypokalaemia or hypernatremia).

## 3 DISCUSSION

Here, we reported on the erroneous administration of GHB rather than BHB in a patient with PA and cardiomyopathy following hospitalization in our hepatology and gastroenterology unit for LT. When the clinician decided to prescribe the ketone body BHB, our hospital's computerized prescription system automatically suggested the referenced GHB. In contrast, BHB is administered as an extemporaneously prepared oral formulation, and therefore was not referenced in the computerized prescription system. Furthermore, several community pharmacies contacted us because recently discharged patients had presented a hospital prescription for GHB instead of the non‐referenced BHB (data not shown). Although the two molecules have different regimens and administration modes, medication errors continue to occur; we believe that healthcare professionals should be more aware of this risk.

The present patient report raises the issue of how orphan pediatric diseases should be managed and monitored from a pharmacovigilance standpoint; treatment may be based on clinical trials with advanced therapy medicinal products, off‐label use of repositioned medications and extemporaneous preparations. Clinical trials allow academics and pharmaceutical companies to develop orphan drugs while allowing patients to benefit from an innovative treatment. Nevertheless, the treatment of rare pediatric diseases with repositioned or extemporaneous prepared medicines is difficult to implement. Given the rarity of certain inborn errors of metabolism, safety and pharmacovigilance data on treatments with orphan drugs and investigational medicinal products are still scarce. A disease is considered to be rare if it affects fewer than five in 10 000 people (according to the European Medicines Agency's definition) or fewer than 200 000 in the United States (according to the American definition). The European Medicines Agency and the United States Food and Drug Administration both encourage academics and pharmaceutical companies to research and develop medicines for rare diseases orphan drug development via fast‐track authorization programmes, orphan drug designations, and the Priority Medicines scheme, for example.^10^, ^11^, ^12^, ^13^, ^14^, ^15^ In addition to the abovementioned clinical trials of advanced therapy medicinal products, pediatric patients with rare diseases can also receive off‐label treatments or extemporaneous preparations in which the drug's regimen and pharmaceutical formulation are adapted for pediatric use. Although the recent medical literature emphasizes the fact that the use of computerized prescription systems can decrease the frequency of prescription errors and iatrogenic adverse events and increase the safety of drug prescriptions, we consider that the system correction algorithm can sometimes generate errors ‐ especially in the setting of rare diseases. Indeed, recent literature on medication errors linked to a computerized physician order entry system emphasized the need to understand and then present the causes of these errors.^16^, ^17^, ^18^, ^19^, ^20^, ^21^, ^22^ Since PA is a rare inborn error of metabolism with a small number of patients, some treatments are not referenced and not well known—resulting in medication errors. In the present case, the similarity between the two drugs' names (“BHB” vs “GHB,” or “3‐hydroxybutyrate” vs “4‐hydroxybutyrate”) increases the risk of confusion and the likelihood of automatically approving a computerized prescription of GHB rather than nonreferenced BHB. In order to prevent this type of prescribing error by healthcare professionals, our hospital pharmacy has produced an information sheet outlining the differences between BHB, GHB, and sodium oxybate.

## 4 CONCLUSION

Although there are many incentives for orphan drug development in Europe and the United States, treatments for rare pediatric diseases are still limited. Lack of knowledge regarding the management of rare pediatric diseases remains the principle cause of medication prescription errors and misuse.

Although an electronic prescribing system should improve prescribing safety and decrease prescribing errors, the present case and other recent cases underline the fact that medication errors can still occur—especially for nonreferenced, extemporaneous preparations, and infrequent prescriptions. Prescriptions should always be accompanied by an information sheet for healthcare professionals that specifies the nature of the medication and its mode of administration.

## CONFLICT OF INTERESTS

All authors declare that they have no conflicts of interest. This work was academic‐led. It was carried out by clinicians and pharmacists independently of any pharmaceutical company. The authors did not receive any specific private‐sector funding for the authorship and/or publication of this case report.

## INFORMED CONSENT

The present case report did not involve preclinical studies in animals or clinical studies. Under French legislation, approval by an independent ethics committee and the provision of patient consent were therefore not required.

## ANIMAL RIGHTS

This article does not contain any studies with human or animal subjects performed by the any of the authors.

## DETAILS OF THE CONTRIBUTION OF INDIVIDUAL AUTHORS

Acquisition, analysis, and interpretation of data: C.T.‐D., P.D.L. Clinical work and treatment supervision: F.M., J.B., M.O., A.B., C.W., S.G.‐L., J.B.A., F.L., S.R., P.D.L. Pharmaceutical review: J.S., S.C., P.H.S. Drafting of the manuscript: C.T.‐D., P.D.L. Critical revision of the manuscript: all authors.
